# The effect of a multidisciplinary approach for smoking cessation in patients with Crohn’s disease: Results from an observational cohort study

**DOI:** 10.18332/tid/119161

**Published:** 2020-04-02

**Authors:** Pierachille Santus, Dejan Radovanovic, Davide Raiteri, Stefano Pini, Giuseppe Spagnolo, Giovanni Maconi, Maurizio Rizzi

**Affiliations:** 1 Division of Pulmonary Diseases, Department of Biomedical and Clinical Sciences (DIBIC), Università degli Studi di Milano, Milan, Italy; 2 Department of Medical and Surgical Physiopathology and Transplantation, Università degli Studi di Milano, Milan, Italy; 3 Division of Gastroenterology, Department of Biomedical and Clinical Sciences (DIBIC), Università degli Studi di Milano, Milan, Italy

**Keywords:** depression, smoking cessation, anxiety, Crohn’s disease, nicotine

## Abstract

**INTRODUCTION:**

Cigarette smoking is the most important risk factor for Crohn’s disease (CD). The effectiveness of smoking cessation programs (SCPs) in patients with CD is still poorly understood.

**METHODS:**

This was a retrospective, observational, single-centre, cohort study of 136 active smokers with mean age 55 years (SD=11), 58% males, including 27 (19.8%) patients with CD who entered the multidisciplinary SCP of the Luigi Sacco University Hospital of Milan from January 2017 through January 2019. A pulmonologist was responsible for the clinical and pharmacological management, while a psychiatrist and a psychologist conducted the counselling and assessed the motivation to quit, anxiety and depression using the Brief Psychiatric Rating Scale (BPRS) and the nicotine dependence with the Fagerström test. Patients were defined as quitters after 12 months.

**RESULTS:**

Demographic and clinical characteristics, and Fagerström score, did not differ in patients with and without CD. At baseline, patients with CD had a higher BPRS (median: 27, IQR: 22–32; vs 25 and 22–28.5; p=0.03), and a lower motivation to quit score (median: 10, IQR: 9–13; vs 14 and 12–15; p<0.001). After 12 months, the quitting rate of smokers with CD was significantly lower (14.8% vs 36.7%; p<0.022) and the chance of quitting was negatively associated with the baseline BPRS (r=-0.256; p<0.003). Varenicline and nicotine replacement therapy tended to be less effective in patients with CD.

**CONCLUSIONS:**

The lower efficacy of SCPs in patients with CD might be secondary to a higher prevalence of anxiety and depression. Psychological issue recognition and support should be enhanced to increase SCP effectiveness in CD.

## INTRODUCTION

Crohn’s disease (CD) is a chronic idiopathic inflammatory bowel disease (IBD). Several factors have been implicated in the development of CD, including genetics and environmental exposure^1^. Cigarette smoking is the most important independent risk factor for CD, being associated with disease development, poorer prognosis, worse quality of life and increased rate of flare-ups and hospital admissions, as well as a higher need for corticosteroids and immunomodulators^2-4^. The risk of CD exacerbation has been found to be proportional to the number of cigarettes currently smoked by patients^5^. Smokers also have a greater risk of perforating complications and fistulisation, of undergoing surgery and of recurrence after a surgery intervention^6-11^. A recent large observational study showed how a smoking habit in patients with CD is associated with higher corticosteroid burden, higher corticosteroid dependence and also a higher risk of intestinal surgery when compared with quitters^12^.

The harmful effects of cigarette smoking can be explained by different pathogenetic processes, such as an intense release of inflammatory cytokines, with consequent immune cell recruitment, and the production of reactive oxygen species coupled with a lessened antioxidant capacity^13^.

Despite these premises, the percentage of smokers in patients with CD remains high, representing a consistent social and healthcare burden^2^; moreover, many of these patients still underestimate the link between smoking and disease worsening, which reduces the motivation to quit^14,15^.

Smoking cessation programs (SCPs), relying on both pharmacological therapy and psychological counselling (including group behavioural therapy) to improve motivation and coping skills, have been proven effective in increasing quitting rates in the general population^16,17^, with success rates ranging from 32% to 36%^18^ (with better results for inpatients^19^ than for outpatients20), greatly surpassing cessation rates obtained via simple physician advice (3–6%)^21^. However, only limited data are available on the efficacy of SCPs in patients with CD^2,5^. Smoking cessation, being a disease modifier, represents an intervention of paramount importance in patients with CD, and should be considered a priority in all CD smokers^4^ as it may result in a significant improvement in disease activity, the need for surgery, and disease-related costs^22^. To date, however, available studies have shown low quit rates in smokers with CD compared to the general population, and the reason for such a difference has not been completely understood, thus limiting physicians’ interventional capabilities.

The aim of the present study was to compare the effectiveness of a multidisciplinary SCP in patients with and without CD and to explore the factors related to quitting failure in patients with CD.

## METHODS

We performed a retrospective, observational, single-centre, cohort study of active smokers who entered the outpatient multi-disciplinary SCP of the Luigi Sacco University Hospital of Milan, Italy, from January 2017 through January 2019. All patients entering the SCP were consecutively enrolled, and no strict eligibility criteria were applied to reflect real-world practice conditions. The study sample included patients affected by CD who had been referred to our SCP by our Division of Gastroenterology. The study was approved by the local ethics committee (2018/ST/169), conducted following the principles of the Declaration of Helsinki 1975 and written informed consent was obtained from all patients. Due to the retrospective nature of the study, the investigators did not have any role in dosing the treatments or in monitoring the exhaled carbon monoxide (CO) levels. All procedures were conducted following the local standard operating procedures in accordance with the principles of good clinical practice.

At baseline, sociodemographic and clinical characteristics were collected. A psychiatrist and a psychologist assessed the level of anxiety and depression by means of the Brief Psychiatric Rating Scale (BPRS)^23^, the motivation-to-quit score^24^ and nicotine dependence with the Fagerström test^25,26^. A pulmonologist monitored the exhaled CO by means of a portable analyser (EC50 Smokerlyser Bedfont Instruments; Kent, UK), and was responsible for the patients’ clinical and pharmacological management. Patients were followed up for 12 months during which they attended group psychological counselling once a week for the first month and were clinically reassessed by the pulmonologist on the 3rd, 6th and 12th month. Suitable patients were also prescribed pharmacological therapy with either varenicline^27^ or nicotine replacement therapy (NRT) through electronic nicotine delivery systems or nicotine transdermal patches^28^, according to clinical and smoking history, psychiatric evaluation and taking into account drug interactions with patients’ chronic therapy. Every visit consisted of a clinical assessment, and a pharmacological adjustment if considered necessary by the treating physician. Treatment compliance, together with exhaled CO, were also checked. We decided to consider a value of 6.5 ppm CO as the cut-off at every check-point to define a quitter as this value has been demonstrated to have satisfactory specificity and sensitivity to distinguish patients exposed to tobacco smoke^29^. A quitter was thus defined as a patient with an exhaled CO value of ≤6.5 ppm at every follow-up visit (i.e. after 3, 6 and 12 months)^29^. During the follow-up period, patients were engaged in single and group meetings with both the psychiatrist and the psychologist, before being examined by the physician. Psychological and motivation-to-quit counselling and support were provided at each visit, based both on an individual and group therapeutic approach, guided by the psychologist.

### Statistical analysis

Statistical analysis was performed with SPSS version 11.5 (SPSS Inc Chicago IL, USA). Variables were expressed as median and interquartile range (IQR) or means with standard deviation (SD), according to their distribution assessed with the Shapiro-Wilk test. Student’s t-test for independent groups, chi-squared, Mann-Whitney or exact Fisher’s tests were used to compare patients with and without CD, or quitters and non-quitters, as appropriate. Relationships between variables were assessed by means of linear regression analysis. Tests were two-sided and statistical significance was set at p<0.05. The STROBE reporting checklist was applied to describe and discuss the results.

## RESULTS

A total of 158 patients were enrolled in the study (62% males). Twenty-two were lost to follow-up at the end of the study: 5 patients had an acute respiratory failure leading to hospitalization, 10 patients left the program due to working issues, 5 patients underwent major cardiac surgery, and two moved to another city. In all, 136 patients (58.1% males; mean age 55 years, SD=11) formed the study sample. Patients had smoked a median of 40 (IQR: 30–50) pack-years and had a high level of dependence on nicotine, while 34.6% reported a previous attempt to quit (Table 1). Twenty-seven patients (19.8%) had CD, with a median age at diagnosis of 40 years (IQR: 29–52); demographic parameters, education level, Charlson index^30^, smoke history, and Fagerström score did not differ in patients with and without CD (Table 1). At baseline, patients with CD had a significantly higher BPRS (median: 27, IQR: 22–32; vs 25 and 22–28.5; p=0.03), and a lower motivation-to-quit score (median: 10, IQR: 9–13; vs 14 and 12–15; p<0.001) (Table 1).

**Table 1:** Baseline patients’ demographic and clinical characteristics. Data are reported for the whole study sample and for patients with and without Crohn’s disease (CD)

*Characteristics*	*Whole sample (N=136)*	*No CD (N=109), a*	*CD (N=27), b*	*p (a vs b)*	
**Demographic features**					
Males, n (%)	79 (58.1)	63 (57.8)	16 (59)	0.535	χ^2^
Age (years), mean (SD)	54.6 (10.8)	55 (10.8)	52.9 (11)	0.789	t-test
**Education level**					
Primary school, n (%)	8 (5.9)	7 (7.4)	1 (4.2)		
Secondary school, n (%)	31 (22.8)	25 (26.6)	6 (25)	0.403	χ^2^
High school, n (%)	58 (42.6)	43 (45.7)	15 (62)		
College, n (%)	21 (15.4)	19 (20.2)	2 (8)		
**Smoking and health status**					
Charlson index, median (IQR)	2.00 (1–3)	2 (1–3)	2 (1–2)	0.14	Mann-Whitney
BPRS, median (IQR)	25 (22–31)	25 (22–28.5)	27 (22–32)	**0.03**	Mann-Whitney
Age started smoking, median (IQR)	17 (15–20)	16 (14–20)	17 (15–18)	0.568	Mann-Whitney
Age at Crohn diagnosis, median (IQR)	40 (29–52)	n/a	40 (29–52)	n/a	
Cigs smoked/day, median (IQR)	20 (15–23)	20 (15–25)	15 (10–20)	**0.0 26**	Mann-Whitney
Pack-years, median (IQR)	40 (30–50)	40 (30–50)	35 (30–45)	0.299	Mann-Whitney
Previously attempted to quit, n (%)	47 (34.6)	38 (34.9)	9 (33.3)	0.536	χ^2^
Exhaled CO (ppm), mean (SD)	17.6 (7.2)	17.8 (7.2)	16.5 (8)	0.983	t-test
Fagerström score, median (IQR)	8 (6–9)	8 (6-9)	8 (6–9)	0.907	Mann-Whitney
Motivation to quit score, median (IQR)	13 (12–15)	14 (12–15)	10 (9–13)	**<0.001**	Mann-Whitney
**Pharmacological treatment**					
None, n (%)	13 (9.6)	10 (9.2)	3 (11.1)	0.498	χ^2^
Varenicline, n (%)	80 (58.8)	62 (56.9)	18 (66.7)	0.241	χ^2^
NRT, n (%)	43 (31.6)	37 (33.9)	6 (22.2)	0.174	χ^2^
**Outcome**					
Quitters, n (%)	44 (32.4)	40 (36.7)	4 (14.8)	**0.022**	χ^2^

Significant differences are highlighted in bold. BPRS: Brief Psychiatric Rating Scale. NRT: nicotine replacement therapy. SD: standard deviation. IQR: inter quartile range. Cigs: cigarettes.

### Study outcome

**Figure 1:**
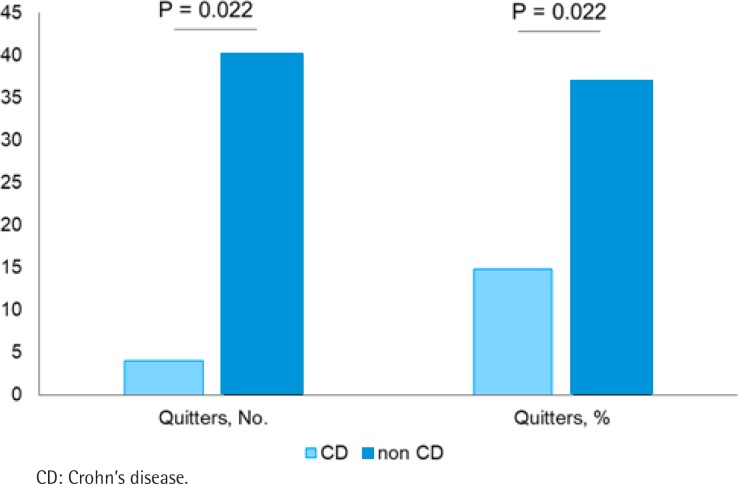
Study outcome. Proportion of quitters in patients with Crohn’s disease (light blue) and without Crohn’s disease (dark blue); data are presented in absolute values (patients) and percentage relative to the whole study sample

At the end of the follow-up, 44 (32.4%) patients quit smoking. The quitters among patients with CD were significantly less compared with patients without CD (14.8% vs 36.7%; p<0.022) (Figure 1).

### BPRS and motivation to quit

Compared with patients that quit at the end of the follow-up, patients that failed to quit had a significantly higher BPRS scale (27.8 vs 24.5; p=0.004) and tended to have a lower motivation-to-quit score (12.8 vs 13.7; p=0.062). The chance of smoking cessation was in general negatively associated with the BPRS scale (r=-0.256; p<0.003) and with the Fagerström score (r=-0.222; p=0.01).

**Figure 2:**
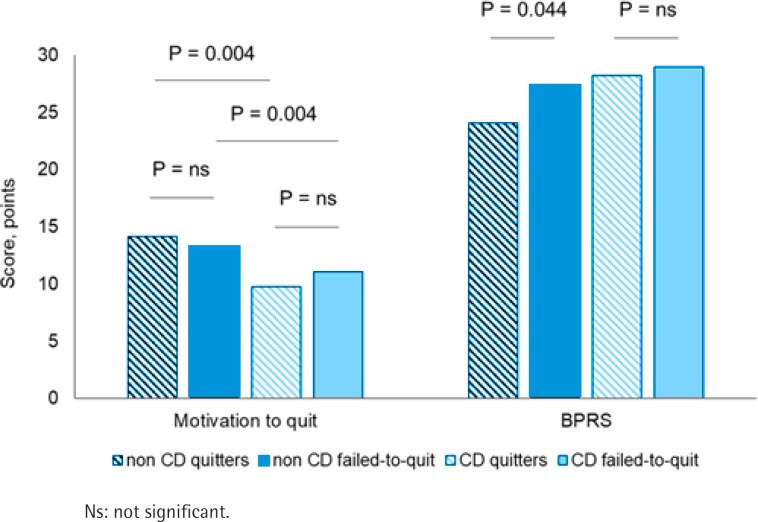
Motivation to quit and BPRS distribution in patients with and without Crohn’s disease. The motivation to quit and the BPRS scores are reported for quitters and non-quitters in patients with Crohn’s disease (light blue striped and full columns, respectively) and without Crohn’s disease (dark blue striped and full columns, respectively)

At baseline, quitters without CD had a significantly higher motivation-to-quit score compared with patients with CD (14.2 and 9.7; p=0.004) (Figure 2). Patients without CD that quit smoking at the end of the follow-up had a significantly lower baseline BPRS score (24.1 and 27.4; p=0.044). The same was not true for patients with CD (Figure 2).

### Pharmacological treatment

The proportion of quitters and quitting failure in the whole study sample did not differ depending on the treatment (9.1% vs 9.8%; 31.8% vs 31.5%; 59.1% vs 58.7%; for no treatment, NRT and varenicline, respectively; p=0.992).

**Figure 3:**
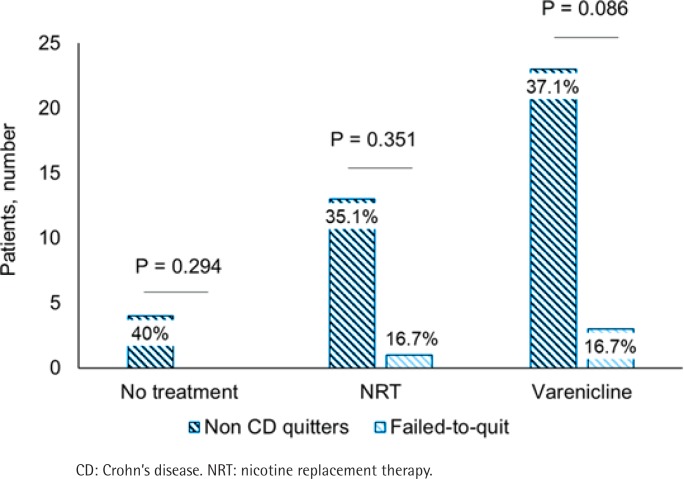
Treatment response in patients with and without Crohn’s disease. The absolute number and the proportion of patients (percentage) that quit at the end of the follow-up period for each treatment regimen are reported for patients with Crohn’s disease (light blue striped columns) and without Crohn’s disease (dark blue striped columns)

No significant difference could be observed in the proportion of patients that received either no treatment, NRT or varenicline between patients with CD and the rest of the study sample (Table 1). Among patients with CD, the proportion of treated patients that quit at the end of the follow-up was less compared with patients without CD, for all treatment regimens (Figure 3).

## DISCUSSION

The main findings of the present study can be summarized as follows: 1) compared with other participants, smokers with CD attending a multidisciplinary SCP had a significantly lower chance to succeed in quitting the smoking habit; 2) a higher proportion of smokers with CD are characterized by anxiety and depression, associated with a lower motivation to quit; and 3) compared with treated smokers without CD, patients with CD exposed to a pharmacological treatment appeared to have less success in quitting smoking at the end of the follow-up.

Many studies strongly support the effectiveness of smoking cessation in modifying the course of CD, with significant reduction in risk of flare-ups, need for surgery^5,6^ and better response to immunomodulating therapy^31^. Twelve months after quitting, risk of CD exacerbations and need for immunosuppressive therapy in quitters were comparable to those of non-smokers and inferior to those of active smokers^2^. However, very few studies have evaluated the effectiveness of a multidisciplinary SCP in patients with Crohn’s disease. A previous interventional study by Cosnes et al.^2^ found lower quit rates among patients with CD compared with the general population (about 12%). A more recent multicentre prospective interventional study, TABACROHN^32^, reported higher success rates, with 23% of 408 active smokers with CD labelled as smoking-free after a median 9-month follow-up.

The low quit rate of smokers with CD found in our study, despite the adoption of multidisciplinary SCP, is comparable to data by Cosnes et al.^2^. The difference with TABACROHN^32^ may be partially due to very different demographic and clinical characteristics of the patients enrolled in the study. In fact, smokers with CD in the TABACROHN study had a median age of 41 years (IQR: 32–48) and had a smoking history of 13 (6–25) pack-years, while our patients with CD were older, and had, on average, a heavier smoking history. Moreover, our findings are difficult to compare with the study of Nunes et al.^32^, due to differences in both study definitions and the interventional strategies adopted. First, in the TABACROHN study, patients were defined as quitters after self-reporting being completely smoking-free for at least one week and were then moved to the relapse group if they had resumed smoking in the subsequent follow-up; at the time of the publication, quitters had a median of 9 months of smoking-free follow-up. Conversely, we defined patients as quitters after a 12-month smoking-free follow-up, checking the exhaled CO to confirm the cessation of smoking habit. Second, in the TABACROHN study the smoking cessation strategy varied according to each centre’s clinical practice, with only 63% of the patients that were willing to quit assisted by a non-gastroenterologist physician (mostly a pulmonologist), and only 12% of patients having received pharmacological therapy^32^.

To date, factors associated with a low success of SCPs in patients with CD are still mostly undefined. The interventional study by Cosnes et al.^2^ found that the physician in-charge, previous intestinal resections, high socioeconomic status and use of oral contraceptives were independent predictors of smoking cessation, while other demographic characteristics, disease duration and smoking history were not significantly associated with the program’s outcome^2^. A survey conducted in 2001 showed similar attitude towards smoking cessation between smokers with and without CD, suggesting that factors not directly related to CD were more important in the willingness to quit^33^. In the TABACROHN study, no predictor of quit smoking was found, but good cessation rates in spite of inhomogeneous pharmacological support and involvement of experts suggest that counselling and the patient–physician relationship were the most effective tools to promote smoking cessation^32^. To our knowledge, this is the first study that found a lower quit smoking rate together with a higher incidence of anxiety and depression in patients with CD, confirming the importance of patients’ psychological burden reported in previous literature^4^.

Epidemiological data show that a significant percentage of patients with IBDs are affected by depression and/or anxiety disorders, particularly those with active disease^34^. On the other hand, smokers with depression are known to be less successful in attempts to quit^35,36^. In fact, nicotine intake improves depressive symptoms by modulating the stress response mediated by nicotine acetylcholine receptors, thus influencing a wide range of neurotransmitters, including 5-HT, dopamine, GABA, and glutamate. Accordingly, smoking cessation has been shown to precipitate depressive symptoms^37^. We thus hypothesize that the lower efficacy of a multidisciplinary SCP in patients with CD may be secondary to a higher prevalence of anxiety and depression^34^, which may drive increased nicotine consumption and expose patients to a greater risk of symptoms worsening after withdrawal^35^. This hypothesis is corroborated by our finding that the BPRS score at baseline was negatively correlated with a successful outcome after an SCP.

Differently from previous reports^30^, the majority of the patients participating to our study (>90%) were given a pharmacological treatment for smoking cessation, despite the absence of healthcare cost coverage for both varenicline and NRT; this may suggest quite a high motivation for quitting the smoking habit in patients enrolled in our SCP. Although the proportion of patients treated with either therapy, in the CD and non-CD groups, were not significantly different, in the present study each pharmacological treatment appeared to be less efficacious in patients with CD who tended to have much lower quitting rates both with varenicline and NRT. The latter finding might be explained by a reduced adherence to pharmacotherapy for smoking cessation^38^, which may be more frequent in patients with CD, already exposed to a higher daily medication burden. However, the adherence to the pharmacological therapies introduced during the SCP could not be assessed retrospectively, and this represents a limitation of the study.

### Limitations

We are aware that the present study has other important limitations that should be highlighted. The number of patients affected by CD is limited, reducing the statistical power and preventing us from making a statistical comparison of demographic and clinical characteristics between quitters and non-quitters within the CD group. This limited our ability to investigate factors that may be related to smoking cessation in these particular group of patients. Our findings should be therefore validated and further explored by prospective studies involving a larger population of patients with CD. Nonetheless, the studied CD population may not reflect the proportion normally found in smoking cessation clinics, but is due to the structure of our teaching hospital and to the local standard procedures; the study is thus characterized by a selection and referral bias, making the generalizability of the results less expected. The availability and reimbursement of NRTs and varenicline may also vary from country to country, being an important confounding factor when comparing our results with other studies.

Moreover, the duration of the follow-up could be ideally longer than 12 months to confirm the long-term effectiveness of SCP and the incidence of smoking relapse; however, 12 months appears to be the most common follow-up duration in studies included in a Cochrane review investigating the role of physician advice in smoking cessation^21^.

## CONCLUSIONS

In a population of smokers attending a multidisciplinary SCP, the success in quitting cigarette smoking was less likely in patients with CD; we also found that patients with CD had a higher prevalence of anxiety and depression, associated with a lower motivation to quit; we speculate that these psychological traits might have a role in jeopardizing the efficacy of the pharmacological treatments proposed for smoking cessation.

In view of the importance represented by quitting the smoking habit in patients with CD, we suggest here some interventions that should prove useful to improve disease control and patients’ disability: 1) primary prevention programmes to increase awareness of the role of smoking in CD, in consideration of widespread lack of knowledge about this topic in patients^14^; 2) psychological issue recognition and personalized counselling in CD smokers, due to high incidence of anxiety and depression in this population and its link to smoking; and 3) facilitated access of smokers with CD to SCPs with expertise in CD, because of the specific challenges posed by smoking cessation in these patients.
